# Dysregulation of IRP1-Mediated Iron Metabolism Causes Gamma Ray-specific Radioresistance in Leukemia Cells

**DOI:** 10.1371/journal.pone.0048841

**Published:** 2012-11-14

**Authors:** Kurtis J. Haro, Aneesh Sheth, David A. Scheinberg

**Affiliations:** 1 Molecular Pharmacology and Chemistry Program and Leukemia Service, Memorial Sloan-Kettering Cancer Center, New York, New York, United States of America; 2 Weill Cornell Graduate School of Medical Sciences, Department of Pharmacology, New York, New York, United States of America; Faculté de médecine de Nantes, France

## Abstract

Iron is required for nearly all organisms, playing important roles in oxygen transport and many enzymatic reactions. Excess iron, however, can be cytotoxic. Emerging evidence suggests that radioresistance can be achieved in lower organisms by the protection of proteins, but not DNA, immediately following ionizing radiation (IR) exposure, allowing for improved DNA repair. One potential mechanism for protein protection is controlling and limiting the amount of free iron in cells, as has been demonstrated in the extremophile *Deinococcus Radiodurans*, reducing the potential for oxidative damage to proteins during exposure to IR. We found that iron regulatory protein 1 (IRP1) expression was markedly reduced in human myeloid leukemia HL60 cells resistant to low linear energy transfer (LET) gamma rays, but not to high LET alpha particles. Stable knockdown of IRP1 by short-hairpin RNA (shRNA) interference in radiosensitive parental cells led to radioresistance to low LET IR, reduced intracellular Fenton chemistry, reduced protein oxidation, and more rapid DNA double-strand break (DSB) repair. The mechanism of radioresistance appeared to be related to attenuated free radical-mediated cell death. Control of intracellular iron by IRPs may be a novel radioresistance mechanism in mammalian cells.

## Introduction

Iron is essential for nearly all living organisms, playing a critical role in oxygen transport in multicellular organisms, as well as in metabolism and various enzymatic processes. Many enzymes use iron as a co-factor to catalyze redox reactions owing to its readily interchangeable ferrous (Fe^2+^) and ferric (Fe^3+^) states, serving as either an electron donor or an electron acceptor [1]. As a consequence, this makes iron one of the most reactive metals in cells, allowing it to undergo Fenton and Haber-Weiss reactions whereby free intracellular ferrous irons can react with hydrogen peroxide (H_2_O_2_) and other cellular peroxides to produce ferric iron, hydroxide (OH^−^), and the highly-damaging hydroxyl (^•^OH) radical. Although iron is essential to many reactions, free iron can be detrimental to cells and must be sequestered and stabilized within the cell in order to prevent these undesirable reactions [2]. Mammalian cells have therefore developed a rapidly responsive iron sequestration system achieved primarily through the function of two key gene products, Iron Regulatory Protein (IRP) 1 and 2. IRPs control the translation of the mRNA of genes involved in the uptake and sequestration of iron by binding iron responsive elements (IRE) in the 5′ and 3′ untranslated (UTR) regions of nascent mRNAs [3], [4]. Key targets of IRPs in circulating hematopoietic cells are transferrin receptor 1 (TfR1), which imports extracellular iron by receptor-mediated endocytosis [5] and the ferritin heavy and light chains (FHC and FLC). The latter proteins combine to form 24 subunit homo- or hetero-polymeric protein structures that catalytically convert labile ferrous iron to a protein-bound, hydrated ferric state where it cannot participate in dangerous Fenton reactions [6]. By controlling expression of these gene products, IRPs allow the cell to maintain intracellular iron homeostasis and sequester excess iron in a non-toxic state. During times of limited iron availability, IRPs bind the 5′ UTR of FLC and FHC and the 3′ UTR of TfR1, thereby increasing intracellular iron availability. Conversely, under conditions of iron excess, IRPs will undergo conformational changes that no longer allow them to bind IREs. Under these conditions, IRP1 becomes a cytosolic aconitase [7] while IRP2 undergoes ubiquitin-mediated degradation [8]. Thus, intracellular iron excess results in increased FHC & FLC protein synthesis and reduced TfR1 protein synthesis, bringing iron levels back to homeostatic levels.

An emerging body of evidence indicates that radioresistance in prokaryotes and other lower organisms is associated with substantially reduced protein oxidation immediately following ionizing radiation (IR) exposure, leading to improved DNA repair, despite nearly uniform induction of DNA double-strand break (DSB) formation per unit dose and base pair in IR-sensitive and IR-resistant cells [9], [10], [11], [12]. In one of the most extensively studied radioresistant microorganisms, *Deinococcus Radiodurans*, proteins are thought to be protected following IR due to the unusually high manganese∶iron ratio, relative to IR-sensitive bacteria. Similarly, many studies have highlighted the potential difficulties cells may face when utilizing iron. For example, metal-containing enzymes are inactivated by hydrogen peroxide via Fenton chemistry when iron is the co-factor, but not if other transition metals are co-factors [13]. Moreover, the free radical superoxide (^•^O_2_^−^), another product of IR, is relatively non-reactive towards DNA directly but highly reactive to [Fe-S] clusters in proteins, inactivating their function and indirectly oxidizing proximal DNA [14], [15]. Finally, a number of studies have demonstrated that increased ferritin expression can lead to protection against a variety of forms of oxidative stress [16], [17], [18], [19]. Hence, the production of indiscriminately damaging hydroxyl radicals by free iron, protein-bound iron's susceptibility to inactivation by peroxides in various enzymes, and the sensitivity of iron-sulfur clusters to superoxide suggests that limiting the role of iron within the cell via enhanced sequestration by ferritin proteins could lead to a survival advantage during exposure to IR. It has been demonstrated that altering the redox activity of mammalian cells by perturbing the expression of the transcription factor Nrf2 also protected against IR by reducing protein oxidation [20], suggesting that this type of radioprotective mechanism is not exclusive to simpler organisms.

We recently discovered that cells made resistant to IR after exposure to low LET gamma rays, but not high LET alpha particles, had significantly reduced IRP1 transcript levels, leading to increased FLC expression (GSE35372). We therefore hypothesized that reduced IRP1 transcript in low LET-resistant HL60 cells was a mechanism of radioresistance in these cells by protecting proteins, but not DNA, from IR-induced oxidative damage immediately following IR exposure. Here, we present data indicating that increased expression of ferritin proteins by reduced IRP1 expression was associated with low LET-specific radioresistance in the myeloid leukemia cell line HL60. The cells were resistant to ionizing radiation (IR)-induced apoptosis, had reduced intracellular Fenton chemistry, and increased clonogenic survival following low LET gamma rays, but not high LET alpha particles, which kill cells primarily through direct DNA damage that is highly complex and often irreparable [21], [22]. These results indicated that the effect was free radical-mediated. The increased survival was associated with lower protein oxidation and more rapid DSB resolution following IR exposure. Cells with decreased IRP1 expression also demonstrated significant resistance to hydrogen peroxide, but not to staurosporine, indicating apoptotic functions were not perturbed. Iron challenge of cells affected the IR-induced apoptotic response in IRP1- cells, but not wild type cells. We conclude that genetic changes that mimic a high iron environment allowed these human myeloid leukemia cells to produce an intracellular environment with less free iron and thus reduced the cell inactivation potential by oxidative damage during gamma ray exposure.

## Results

### IRP1-targeted shRNA specifically reduces mRNA levels of IRP1 and increases FLC protein expression

HL60 leukemia cell clones resistant to either high LET (RA11) or low LET (RG8) IR were selected and assayed for whole-genome expression by microarray analysis (GSE35372). The ten genes with expression most significantly changed in untreated RA11 and RG8 cells relative to radiosensitive control HL60 cells are listed (Table 1). Of the genes listed, one in particular, ACO1 (IRP1), appeared to have a potentially direct role in a low LET-specific radioresistant phenotype, as down-regulated expression of this gene was observed only in low LET IR-derived radioresistant cells (RG8), but not in high LET IR-derived radioresistant cells (RA11). Other genes whose expression changed significantly did not appear to have known roles in the biology of radioresistance, and thus were not studied at this time. We hypothesized that the reduced IRP1 transcript levels in RG8, but not RA11 cells, could be a mechanism of radioresistance specific to low LET gamma rays, due to its role in controlling intracellular iron content.

**Table 1:** The ten most significant gene expression changes in cells made radioresistant to either alpha particles or gamma rays, relative to HL60 cells as measured by genome-wide microarray analysis.

Alpha IR-derived cells (RA11)		Gamma IR-derived cells (RG8)	
Gene ID	Fold Change (Relative to HL60 cells)	Gene ID	Fold Change (Relative to HL60 cells)
UBB	−5.9	UBB	−10.2
DYSFIP1	69.9	DYSFIP1	27.3
ID3	4.5	DEFA1	−4.2
IL23R	−5.9	LOC728358	−3.2
FOXC1	5.3	DEFA3	−3.4
HSD11B1	−2.6	MAGED1	−18.2
MSRB2	4.9	CMBL	−10.7
PRSSL1	6.4	ACO1(IRP1)	−12.2
CCDC92	4.2	ID3	5.5
CLEC5A	−3.1	SPNS3	−2.7

**Figure 1:**
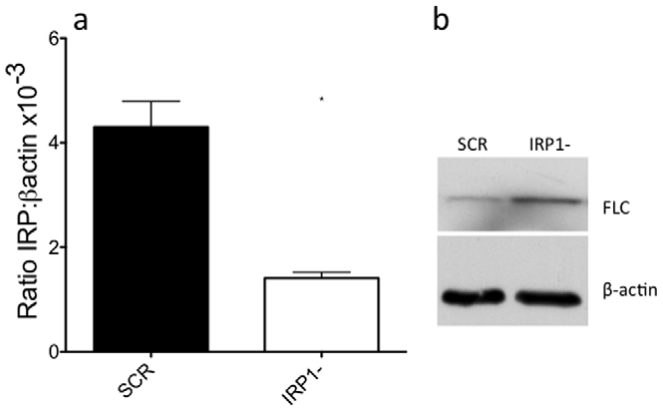
shRNA-expressing viral particles reduce the level of IRP1 mRNA and increase FLC expression. Panel a: following viral infection and selection on puromycin, cells were assayed by qPCR to measure IRP1 transcript levels. Results are plotted as ratio of IRP1 transcript to β actin transcript. Results are mean +/− SEM of three independent experiments. A paired t-test was used to test significance (* = p<0.05). Panel b: cell lysates were normalized by protein content using a Lowry assay and analyzed by western blot analysis with a antibodies against human FLC (representative image).

We sought to model, reproduce, and validate the effect of low IRP1 in cells using RNAi. Following transduction and puromycin selection of non-irradiated HL60 cells stably expressing shRNA targeted to IRP1 (IRP1- cells), we determined the level of IRP1 mRNA using quantitative real-time PCR. Four of five IRP1-targeted shRNA sequences reduced IRP1 transcript levels to less than 50% of control cells selected following infection with non-targeted (scrambled, SCR) shRNA control, relative to beta actin (Fig. S1A). The fifth sequence did not reduce IRP1 transcripts. The most effective sequence (IRP1-.2, dubbed IRP1-) was assayed for IRP1 mRNA levels over several days in culture and found to lower IRP1 mRNA levels to 32% of control virus levels (Fig. 1A). Four different commercial antibodies to human IRP1 were not capable of recognizing native IRP1 in HL60 cells at the appropriate size (although one was capable of identifying IRP1 in Hela cells), and thus IRP1 protein levels could not be determined.

Since loss of IRP1 should lead to higher levels of ferritin protein, we measured the levels of FLC by western blot analysis. FLC expression was increased relative to cells transduced with SCR shRNA cells by an average of 3.6+/−0.9 fold from four independent lysates after normalizing to beta actin (Fig. 1B). These data indicate that stable knockdown of IRP1 increased intracellular ferritin protein levels, as predicted.

### IRP1- cells have significantly decreased reactive oxygen species formation following exposure to hydrogen peroxide

**Figure 2:**
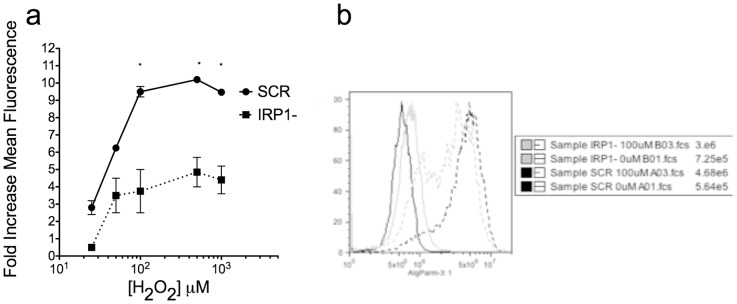
Hydrogen peroxide-induced ROS formation is decreased in IRP1- cells. Panel a: cells were loaded with 100 nM H_2_-DCFDA and then assayed for increases in fluorescence by flow cytometry following indicated doses of hydrogen peroxide, plotted as fold increase over control. Data are mean +/− SEM of three independent experiments. Two-way ANOVA was used to test significance at each dose (* = p<0.05). Panel b: Representative histograms treated with 0 and 100 µM hydrogen peroxide.

Conventional assays to measure iron, such as the ferrozine method and the metal-sensitive calcein fluorescent probes, were not sensitive enough to determine the amount of free intracellular iron in as many as 10^8^ cells. Therefore, we indirectly determined free iron content by assaying for iron-induced Fenton chemistry. The cell-permeant probe 2,7, dichlorohydrofluorescein diacetate (H_2_-DCFDA) becomes trapped inside living cells by esterase activity, and becomes fluorescent upon oxidation [23]. We loaded cells with this probe then measured the increase in fluorescence upon treatment with hydrogen peroxide (H_2_O_2_). While the hydrogen peroxide itself will increase fluorescence in any cell, hydroxyl radicals produced by Fenton chemistry will contribute to brighter overall fluorescence. H_2_O_2_-induced fluorescence was significantly decreased in IRP1- cells compared to SCR cells, suggesting that intracellular free iron is reduced in IRP1- cells (Fig. 2A–B).

### Knockdown of IRP1 reduces IR-induced protein oxidation

**Figure 3:**
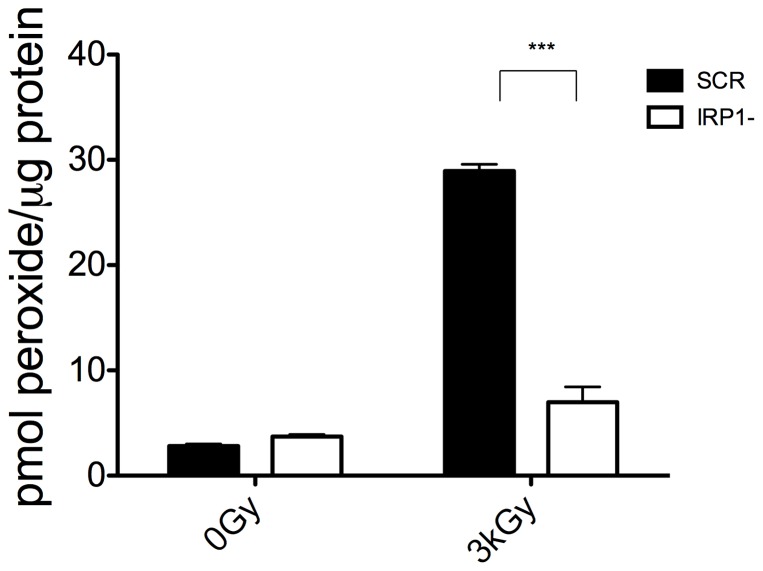
IRP1 knockdown reduces IR-induced protein peroxide content. Immediately following IR exposure at indicated doses, cells were washed and then frozen in −80°C ethanol before processing lysates for protein peroxide content by the PCA-FOX assay. Data are plotted as pmol of peroxide per microgram of protein from a hydrogen peroxide standard curve. Data are mean +/− SEM of a representative experiments performed three times. Student's t-test was used to test significance (* = p<0.05).

There is a strong link between lack of protein oxidation following IR and radioresistance [9], [12], [20]. To assess whether IRP1 knockdown, with resultant reduced reactive intracellular iron, prevented IR-induced protein oxidative damage in HL60 cancer cells, we assayed for protein peroxyl adducts in cells following large doses of gamma rays. Despite the high doses required to achieve measurable signals, we found that IRP1- cells had significantly reduced protein peroxidation compared to control cells (Fig. 3). The level of protein carbonylation relative to untreated controls was also reduced by 30% in IRP1- cells at 3 kGy. These data suggest that knockdown of IRP1 protects cells from protein damage following gamma IR, which could lead to improved cell survival.

### Knockdown of IRP1 reduced IR-induced apoptosis and improves clonogenic survival following low LET IR exposure

**Figure 4:**
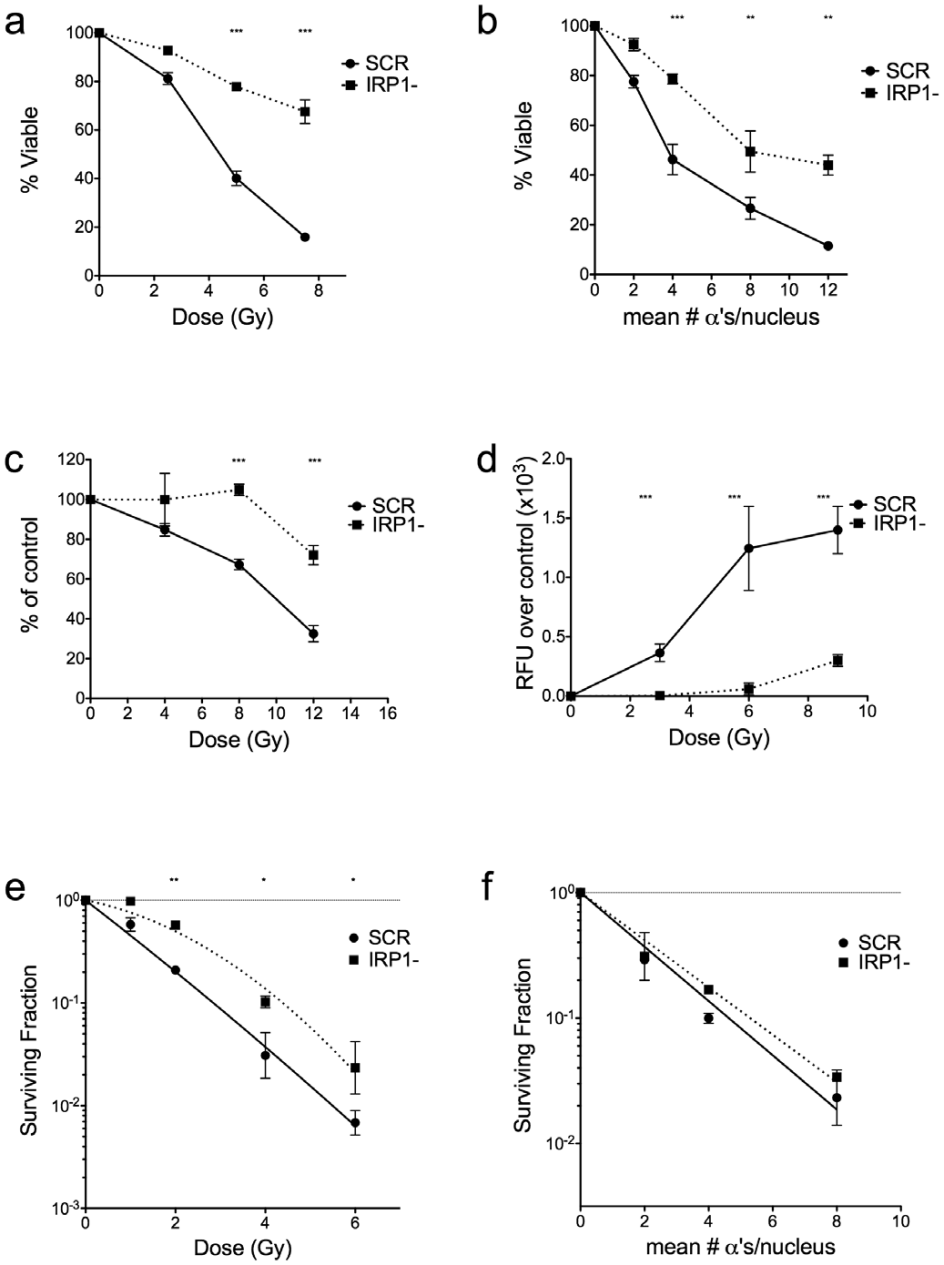
IR-induced apoptosis is reduced and clonogenic survival following low LET IR is improved in IRP1- cells. Cells were exposed to indicated doses of gamma IR (panel a, c, & d) or alpha IR (panel b) and assayed for apoptosis by annexin V & PI staining (panels a–b) at 48 h post-IR, Alamar Blue assay at 48 h post-IR (panel c), or executioner caspase activity at 24 h post-IR (panel d). Data are mean +/− SEM of at least two independent experiments. Two-way repeated measures ANOVA was used to measure statistical significance at each dose (** = p<0.01, *** = p<0.001). Panels e–f: clonogenic survival assays approximately 14 d following gamma IR (panel e) or alpha IR (panel f). Data are mean +/− SEM of three independent experiments. Curve fits compared by a paired t-test were found to be significantly different following gamma IR (p<0.01) but not alpha IR.

To see if knockdown of IRP1 led to changes in IR-induced cell death, we assayed for apoptosis following IR from both gamma rays and alpha particles. IR-induced apoptosis was significantly reduced following both types of radiation at 48 h post-IR in IRP1- cells (Fig. 4A–B), where apoptosis in HL60 cells is near maximal (Fig. S2). These data indicate that reducing IRP1 expression led to less early IR-induced cell death via apoptosis, an important form of cell death in the HL60 cell line. These results were confirmed for gamma IR with the Alamar Blue viability assay (Fig. 4C) and by measuring executioner caspase activity 24 h post-IR (Fig. 4D), where IRP1- cells were found to have a 30% increase in caspase activity at a dose of 9 Gy, compared to a >500% in control cells, indicating that caspase-mediated apoptosis following IR is substantially reduced in IRP1- cells. To ensure these results were not an artifact of this specific shRNA sequence targeted to IRP1, we irradiated HL60 cells transduced with virus expressing all four of the effective IRP1-targeted shRNA sequences, where we found all sequences significantly reduced apoptosis to gamma rays compared to cells transduced with control SCR shRNA (Fig. S1B).

To confirm if knockdown of IRP1 led to reduced mitotic death following IR, we performed clonogenic assays. IRP1- cells had significantly increased cell survival following low LET gamma rays, but showed no significant resistance to high LET alpha particles (Fig. 4E–F). Because alpha particles induce direct DNA damage, which is also more complex and thus more difficult to repair [24], the data suggest that IRP1's role in protein protection or IR-induced apoptosis is only sufficient to protect cells against gamma rays, but cannot repair the complex, multiply-damaged sites (MDSs) alpha particles produce in DNA.

### IRP1- cells have improved DNA DSB foci resolution

**Figure 5:**
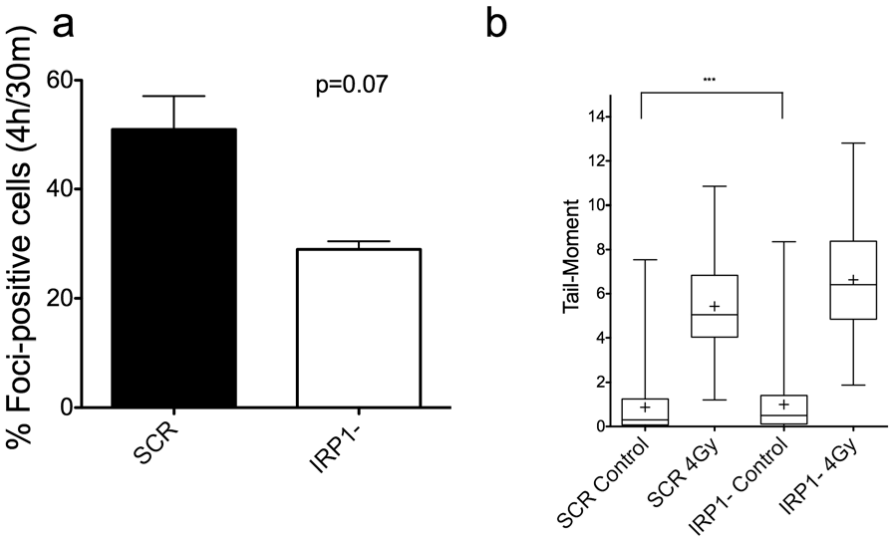
Repair, but not initiation, of DSBs is more rapid in IRP1- cells. Panel a: the increase in the number of foci-positive cells (those containing greater than 10 per nucleus) versus control and 2 Gy irradiated cells were scored at 30 m and 4 h post-IR and are plotted as percent foci-positive at 4 h post-IR versus 30 m. Data are mean +/− SEM from two independent experiments stained simultaneously. Exactly 500 cells were scored per sample. The paired t-test p-value was 0.07. Panel b: cells were irradiated with 0 or 4 Gy gamma rays and immediately assayed for DSB formation by the neutral comet assay. Whiskers represent the 1^st^ and 99^th^ percentiles, and means are indicated by ‘+’ (*** = p<0.001).

To determine if the lack of protein oxidation leads to improved repair of DNA damage following IR, we measured the formation and elimination of phospho-S139 H2A.X (γH2A.X), a biochemical marker of DNA DSBs [25]. While the number of positive cells (defined as nuclei containing 11 or more foci) 30 minutes after 2 Gy gamma IR was higher in IRP1- cells relative to control cells, at 4 h post-IR the number of foci-positive cells in IRP1- cells were lower. Thus, the resolution of IR-induced γH2A.X foci in IRP1- cells was moderately improved relative to control cells (Figs. 5A and S3). These data suggest that more rapid DNA repair may be taking place in IRP1- cells relative to control cells. The initiation of DSB formation immediately following IR was complemented by data from the neutral comet assay, where we observed slightly higher mean and median comet tail-moments in IRP1- cells (Fig. 5B), suggesting that initial DSB formation is not lower in IRP1- cells. The unexpected observation of higher 4n–8n DNA content in IRP1- cells (Fig. S4) explains the slightly elevated number of DSBs in IRP1- cells immediately after IR. Thus, knockdown of IRP1 did not decrease the initial formation of DSBs, as expected.

To ensure that more rapid DNA repair in IRP1- cells was not a result of changes in expression of important DNA repair pathway proteins, we probed for select DNA repair proteins; DNA-PKcs, ATR, and APE1. Our results indicated that, relative to β–actin, the expression of these proteins was similar in wild-type and IRP1- cells (Fig. S5). Thus, the data indicate that expression of these repair proteins *per se* was not responsible for improved survival following IR.

### Radioresistance in IRP1- cells is mediated by a free radical mechanism

**Figure 6:**
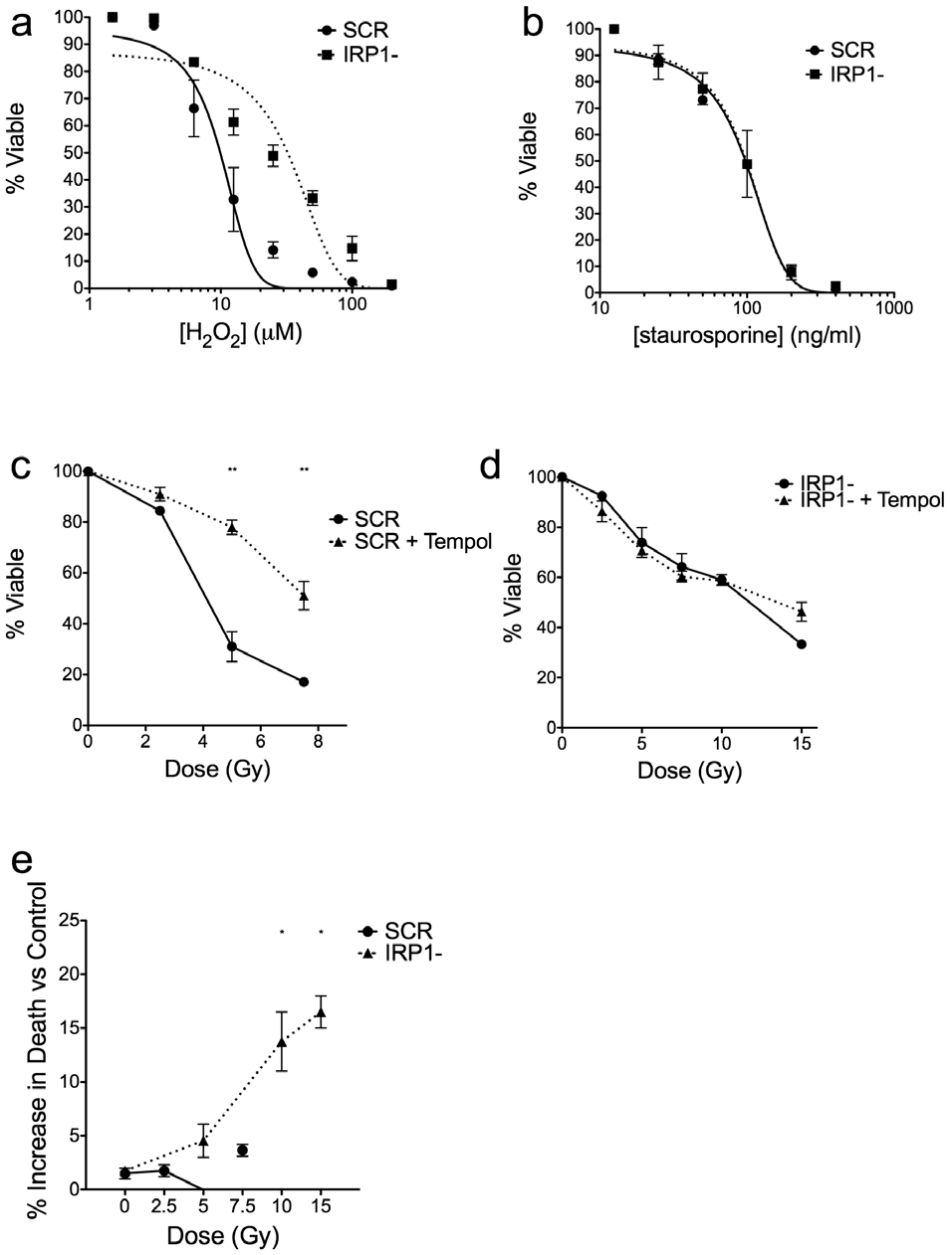
Radioresistance in IRP1- cells is associated with iron availability and a free radical-mediated mechanism. Panels a–b: IRP1- and control cells were treated with indicated doses of chemicals and assayed for apoptosis by annexin V & PI staining at 24 h post H_2_O_2_ treatment (panel a) or 48 h post staurosporine treatment (panel b). Data were fitted to a non-linear variable slope sigmoidal response curve. Panels c–d: cells were treated with 10 mM Tempol for 15 m at 37°C before exposure to indicated doses of gamma rays. Cells were immediately washed and cultured in regular medium for 48 h then assayed for apoptosis by annexin V & PI content. Two-way repeated measures ANOVA with Bonferroni post-tests was used to measure statistical significance at each dose (** = p<0.01). Panel e: cells were treated with 1 mg/mL of purified human apo-transferrin (control) or transferrin for 6 h in serum-free medium and exposed to equitoxic doses of gamma rays (doses were terminated at 7.5 Gy for control cells because cells were 80% dead or more beyond this dose). Cells were immediately washed and placed in regular culture medium and assayed for apoptosis at 48 h by annexin V & PI content. Data were normalized to viability from apo-tansferrin treated cells and analyzed for significance at equitoxic IR doses using a two-way repeated measures ANOVA with Bonferroni post-tests (* = p<0.05). All data are mean +/− SEM from three independent experiments.

While H_2_O_2_ would be expected to damage proteins and DNA, leading to apoptotic death, less free iron should lead to protection of cells because the formation of the highly damaging hydroxyl radicals would be decreased. Whereas cells can catabolize H_2_O_2_ with the enzyme catalase, hydroxyl radicals produced by Fenton chemistry cannot be directly cleared enzymatically and will lead to increased cellular damage, including DSBs. IRP1- cells showed significant resistance to H_2_O_2_ at 24 h post treatment with a shift in IC_50_ of approximately 4-fold (Fig. 6A). Other groups have reported that the lack of mitochondrial iron sufficiency affects mitochondrial function [26]. Since mitochondria play a major role in apoptosis from a variety of stimuli, we treated cells with staurosporine, a cytotoxic molecule not dependent on reactive oxygen species (ROS). Staurosporine induces apoptosis partially through mitochondria [27]. We found that there was no difference in staurosporine-induced apoptosis between cell lines (Fig. 6B). These results indicated that knockdown of IRP1 does not prevent apoptosis generally to cytotoxic agents that kill via apoptosis. Rather, the protection of cells from apoptosis via IRP1 knockdown appears specific to free radical-based cellular insults. We also measured mitochondrial mass versus polarization states in control and IRP1- cells using the cell-permeable dye 5,5′,6,6′-tetrachloro-1,1′,3,3′-tetraethylbenzimidazolylcarbocyanine iodide (JC-1) (mass to polarization ratios of 2.05 and 1.85, respectively). These data also support the notion that radioresistance in IRP1- cells is not due to mitochondrial dysfunction.

To further test a free-radical mediated mechanism of radioresistance in IRP1- cells, we pretreated cells with 4-Hydroxy-2,2,6,6-tetramethylpiperidine 1-oxyl (Tempol) before IR, which has been shown to protect cells and animals from ROS-mediated cytotoxicity, including radiation, due to its ability to quench certain types of free radicals [28], [29]. A non-toxic, 15 m pre-incubation with 10 mM Tempol prior to IR exposure protected wild-type but not IRP1- cells from gamma ray-induced apoptosis (Figs. 6C–D), suggesting that free-radical protection from IR is saturated in IRP1- cells.

Finally, we pre-loaded cells with empty or iron-bound purified human transferrin in serum free medium and irradiated cells with gamma rays. We found that treatment of IRP1- cells with 1 mg/mL of transferrin 6 h prior to IR caused significant increases in gamma IR-induced apoptosis compared to controls cells at equitoxic radiation doses above each cell line's IC_50_ radiation dose (Fig. 6E), relative to apo-transferrin-treated cells (the IC_50_ for IRP1- cells is two-fold higher than wild-type cells, hence the doubling of the IR doses used for IRP1- cells in this assay). Assuming control cells are iron-replete, these data suggest that knockdown of IRP1 mediates IR resistance at least partially through intracellular iron content. Collectively, these data argue that protection against IR-induced oxidative stress related to intracellular iron content is an important factor of radioresistance in IRP1- cells.

Previous studies in our laboratory have indicated that cell cycle perturbations can be an important contributor to radioresistance in HL60 cells (under review). We therefore performed the mitotic index assay on these cells [30]. We found that the dose-dependent mitotic re-entry was similar at 24 h in both cell lines (Fig. S6A). Similarly, the time-dependent checkpoint release following 6 Gy of gamma rays (Fig. S6B) was similar in control and IRP1- cells. These results were confirmed by the micronucleus assay (Fig. S6C–D). Therefore, changes in checkpoint activity were not responsible for the radioresistance we observed in IRP1- cells.

## Discussion

We have demonstrated here a role for perturbations in cellular iron homeostasis as a cause of radioresistance in myeloid leukemia cells via the loss of function of IRP1. The reduction of this regulator of mRNA translation led to increased ferritin expression and reduced intracellular labile iron, which correlated with reductions in IR-induced protein damage, increases in DSB repair, reduced apoptosis, and improved clonogenic survival following IR. These latter results were specific to low LET IR and hydrogen peroxide, both of which elicit their cytotoxic effects primarily through free radicals. The radioresistance was related to intracellular iron, since pre-loading IRP1- cells with excess iron was able to partially revert the radioresistant phenotype.

These characteristics are not a trivial, non-physiologic result of an RNAi-mediated knockdown, because the initial observation of decreased IRP1 transcript levels was made in a cell line made resistant to gamma rays. Importantly, IRP1 reduction was not observed in a cell line made radioresistant by repeated alpha particle exposure, which kills cells via complex DNA damage elicited through direct nuclear traversals of alpha particles. In the gamma resistant RG8 cells (Table 1), we found decreased intracellular Fenton chemistry, overexpression of FLC, and more rapid DSB foci resolution compared to control cells, similar to the results reported here in IRP1- cells. In contrast, none of these characteristics were observed, or were substantially lower, in *alpha particle*-derived RA11 cells. Overall, the data suggest that intracellular iron homeostasis and protection of proteins play a significant role in radioresistance in myeloid leukemia, similar to mechanisms previously described in radioresistant microorganisms. Although alpha particle-induced apoptosis was also reduced in IRP1- cells, clonogenic survival following alpha particles was not significantly improved, confirming that these protective mechanisms are related to the quality of cellular damage, rather than damage *per se*, as alpha particles cause predominantly direct DNA damage that is usually irreparable despite fully functioning cellular repair systems. Although these data are somewhat contradictory, it can be explained by i) the substantially decreased dependence on apoptosis in overall cell death from high LET IR [31], ii) the complex interplay of DNA damage, checkpoint activation, apoptosis, and post-mitotic death following exposure of cells to IR, or some combination thereof.

Furthermore, given the important role of secondary ROS in overall damage to biomolecules in cells during exposure to low LET IR, it is reasonable that we observed down-regulation of the expression of the IRP1 gene in cells chronically irradiated with gamma rays, but not in cells irradiated with high LET alpha particles. However, in those studies we found that both types of resistant cell lines were cross-resistant to the other type of radiation, and that there were many additional perturbations in these resistant cell lines due to many DNA mutations. As a result, we conclude that the loss of IRP1 is one of several potential mechanisms for radioresistance for cells exposed to gamma rays. By studying the role of this gene in radioresistance in isolation, in comparison to isogenic, non-irradiated HL60 cells, we could dissect its function more precisely. Our results presented here agree with the hypothesis that down-regulation of IRP1 protects predominantly against gamma rays, but not high LET alpha particles in assays for clonogenic cell death, and that the effect is likely due to control of iron metabolism and not a general anti-apoptotic phenomenon. Due to the strong evidence that supports sequestration and limitation of intracellular iron as a protective mechanism against IR in some microorganisms [32], our results indicate that a similar mechanism of radioprotection is possible in human cancer cells as well.

Our findings suggest that IRPs and other iron-controlling proteins could serve as targets for cellular radioprotection, such as during nuclear accidents and after detonation of radiation-containing devices. The identification of small molecules that could affect the activity and conformation of IRP proteins could serve as radioprotective agents during incidences of radiation exposure, as such molecules could mimic the downregulaton of IRP proteins and the resultant radioprotective effects described in this work. More studies are warranted to determine if IRP proteins could serve as suitable targets for these purposes.

## Materials and Methods

### Cell Culture

HL60 human myelocytic leukemia cells were obtained from the American Type Culture Collection (ATCC, Mannasas, VA) and were maintained in RPMI medium containing 10 mM HEPES buffer, 2 mM L-glutamine, 10% fetal bovine serum (FBS), 100 µM non-essential amino acids, 100 µg/ml penicillin-G, and 100 µg/ml streptomycin and grown in a 37°C humidified incubator containing 5% CO_2_. Cell densities were maintained at 5×10^4^ to 1×10^6^ cells/ml.

### Development of LET-specific resistant cell clones

HL60 cells were irradiated fifteen times over the course of approximately 150 days with equitoxic, escalating doses at a density of 10^6^ cells in 2 mL of regular medium with either a ^137^Cs source or an ^241^Am source [33] for low and high LET resistant cells, respectively. Unirradiated HL60 control cells were kept in culture throughout the approximately 150 day selection process as a control. Following each round of irradiation, cell viability was monitored by the trypan exclusion technique until >95% cell viability was achieved, after which cells were immediately treated with another round of irradiation. Doses began at levels leading to 90% cell death and were escalated to a dose that achieves approximately 99.9% cell death in naïve HL60 cells. Following the final round of irradiation, individual cell clones from the irradiated progeny and unirradiated HL60 cells were selected after two weeks of growth in semi-solid methylcellulose medium. These colonies were expanded then aliquotted for long-term liquid nitrogen storage.

### Microarray

Total RNA was collected from unirradiated, 8 Gy gamma ray-, or 2 Gy alpha particle-treated cells on three independent days using the Qiagen (Valencia, CA) RNeasy Protect isolation kit per manufacturer's protocol. Transcriptomes were analyzed on Illumina HT-12 microarray plates described in detail in the GEO accession # GSE35372.

### Gamma-H2A.X foci counting

Following IR, cells were fixed in 2% paraformaldehyde (Sigma, St. Louis, MO) for 15 m in the dark, blocked for 30 m with 2% bovine serum albumin (BSA) in phosphate buffered saline (PBS), then incubated for 1 h with a monoclonal antibody anti-phospho-H2A.X (clone JBW301) (Millipore, Temecula, CA) at a 1∶1000 dilution. The slides were extensively washed with PBS, then probed with secondary antibody goat anti-mouse IgG conjugated to AlexaFluor-488 in PBS plus 2% BSA at 1∶1000 dilution for 30 m in the dark. After extensive washing, cells were mounted in ProLong anti-fade mounting solution (Invitrogen) containing 4′,6-diamidino-2-phenylindole (DAPI) and imaged by widefield microscopy (Mirax). Foci size and threshold were adjusted to fit the images and all samples were quantified by automated analysis using Volocity™ with Identical exposure times and threshold settings.

### Neutral Comet Assay

Cells were irradiated on ice at 10^5^ cells/mL in PBS with various doses of gamma IR and immediately processed for DSB formation using the comet assay kit (Trevigen, Gaithersburg, MD) according to manufacturer's protocol via the neutral electrophoresis method. Images were analyzed using CometScore™ (TriTek Corporation, Sumerduck, VA) software. The tail moments were reported for at least 100 cells per sample.

### Annexin V and Propidium Iodide (PI) apoptotic assay

The annexin V apoptosis kit (BD Pharmingen) was used to quantitate early cell death and used according to manufacturer's protocol. 2×10^4^ events were acquired on a C6 Accuri flow cytometer (Accuri Cytometers, Ann Arbor, MI) for each sample.

### Alamar Blue cell viability assay

Cells were irradiated in regular medium at a density of 5×10^4^ cells per mL and plated in quadruplicate in a 96 well plate at 100 µL per well. Cells were incubated at 37°C for 24 h before adding 10 µL per well of Alamar Blue reagent (AbD Serotec, Raleigh, NC). After an additional 24 h at 37°C, fluorescence was measured at 535ex/585em.

### Executioner caspase assay

Cell lysates were prepared in non-denaturing conditions, normalized by protein content, and incubated with fluorogenic substrate Ac-DEVD-AMC (Calbiochem, Darmstadt, GER) at 37°C for 90–120 m and fluorescence was measured at 485ex/535em in a 384 well black plate.

### Alpha particle exposure

The design of the 7mCi ^241^Am source has been described in detail elsewhere [33], [34]. The flux of alpha particles per unit area using the CR-39 plastic etching technique was found to be 198+/−14 particles mm^−2^ from a 0.25 s exposure. All cell samples were settled on the irradiator for 20 m prior to exposure to ensure a uniform cell layer on the mylar cell culture surface. Mean incident LET of the alpha particle is estimated at 132 keV/µm. A 1 Gy nuclear dose of alpha particles is conservatively estimated at four traversals per nucleus, according to the measured nuclear area of HL60 cells and the methods of Charlton and Sephton [35].

### Gamma ray exposure

Cells were exposed to low LET IR by a Shepherd Mark I irradiator (JL Shepherd, Glendale, CA) with a ^137^Cs source at either ∼2 Gy/min at room temperature and to a 37°C standard cell culture incubator or ∼50 Gy/min with a ^60^Co source on ice and immediately processed for protein oxidation.

### Mitotic index assay

At indicated times, cells were fixed and assayed for mitotic index as described [36].

### Micronucleus assay

Following IR treatment, cells were treated with 10 µg/mL cytocholasin B for 20 h under normal cell growth conditions and assayed for micronucleus formation as described previously [37].

### Clonogenic survival assay

Cells were adjusted to ten times plating concentration and gently mixed 1∶10 with aliquots of methylcellulose (Stemcell Technologies) containing 40% methylcellulose, 10% FBS, 2 mM L-glutamine, and 50% RPMI with a blunt ended 16 gauge needle (Stemcell Technologies) before plating 1.1 mL per 35 mm^2^ dish in triplicate for each sample. Following ∼14 days in a 37°C humidified incubator, samples were blinded and colonies enumerated by counting colonies containing greater than 50 cells.

### Measurement of iron-induced reactive oxygen species formation

The production of iron catalyzed reactive oxygen species (ROS) was measured using the ROS-reactive probe 2,7, dichlorohydrofluorescein diacetate (H_2_-DCFDA) according to manufacturer's protocol. Approximately 10^5^ cells were incubated with 100 nM of H_2_-DCFDA in 1 mL of PBS for 30 m at 37°C. Cells were then washed twice with PBS before resuspension in 500 µL of cold PBS containing various concentrations of H_2_O_2_. Cells were incubated on ice for 5 m before acquisition on a flow cytometer. Mean peak fluorescence was recorded for each sample and plotted as fold increase over controls.

### Determination of protein oxidation following IR

Cells were irradiated on ice in PBS at 10^6^ cells/mL and spun down for 5 m at 400 g before freezing in a dry ice ethanol bath. Lysates were prepared in a 10 mM HEPES buffer with protease inhibitors and lysed by homogenization. Lysates were spun in a cold room centrifuge for 10 m at max speed and protein was quantified by Lowry method. Carbonyl content was measured using the Oxyblot kit (Millipore, Temecula, CA) and protein peroxides were measured following precipitation by perchloric acid (PCA, Sigma Chemicals, St. Louis, MO) using the PCA-FOX assay described elsewhere [38]. Data were reported as picomoles of peroxide per microgram of protein from a hydrogen peroxide standard curve. Due to the sensitivity of the assays, high doses were necessary to achieve good signal-to-noise ratios.

### IRP1-targeted shRNA knockdown viral production and infection

MISSION™ targeted shRNA constructs were obtained from Sigma for the ACO1 (IRP1) gene (product # SHCLNG-NM_002197). The primary, pre-validated IRP1-specific sequence used in this study was 5′CCGGGCAGGATTGTTAGCAAAGAAACTCGAGTTTCTTTGCTAACAATCCTGCTTTTTG3′, while the control non-targeted sequence used was 5′CCGGCAACAAGATGAAGAGCACCAACTCGAGTTGGTGCTCTTCATCTTGTTGTTTTT3′. Other IRP1 targeted sequences used in this study to confirm these results were 5′CCGGCCAGGAAAGAAATTCTTCAATCTCGAGATTGAAGAATTTCTTTCCTGGTTTTTG3′, 5′CCGGCGTATATCAAATCACCACCATCTCGAGATGGTGGTGATTTGATATACGTTTTTG3′, 5′CCGGCCTACAAGAAAGCGGAGTCATCTCGAGATGACTCCGCTTTCTTGTAGGTTTTTG. shRNA-containing plasmid constructs were packaged with lentiviral plasmids per manufacturer's protocol using the cell line HEK293T (ATCC, Mannassas, VA). Viral soups were titered using the HIV-1 p24 antigen ELISA assay kit (Zeptometrix, Buffalo, NY). Target cells were incubated overnight with virus at a multiplicity of infection of 10. Cells were then washed and placed in standard growth medium for another 24 h before selection on 5 µg/mL puromycin for ten days with frequent replenishment in standard cell culture conditions before being returned to normal growth medium.

### Quantitative real time polymerase chain reaction

RNA was isolated from ∼10^6^ cells per sample using the RNeasy mini kit (Qiagen) according to the manufacturer's protocol. Between 0.5–1.0 µg of total RNA was used as a template for cDNA synthesis using Superscript III first-strand synthesis kit (Invitrogen) according to manufacturer's protocol. Universal PCR master mix (Roche) was used with IRP1 sequence-specific primers and FAM-labeled probes (product # Hs00158095_m1) for quantitative PCR analysis in a Fast 7500 Real-time PCR system (Applied Biosystems). Expression levels were normalized to β actin primer and probe mixtures (product # HS99999903_m1).

### Western blot analysis

20 µg of cell lysate per lane were run on 4–15% gradient Tris-HCL polyacrylamide gels (Bio-Rad) for 40 m at 180 V. 100 ng of purified human ferritin from liver was used as a positive control (Sigma Chemicals). Proteins were transferred to PVDF membranes for 2 h at 30 V in a Tris-Glycine buffer. Membranes were blocked for 30 m in 4% non-fat milk in tris buffered saline (TBS) before incubation overnight at 4^•^C with mouse monoclonal antibody to FLC (Santa Cruz Biotechnology) in 4% milk in TBS. Membranes were washed twice for 5 m at room temperature with TBS containing 0.05% Tween-20 (TBST) before incubation with goat anti-mouse-HRP secondary antibody (Santa Cruz Biotechnology) and goat anti-rabbit-HRP at 1∶2000 for 2 h at room temperature. Membrane wash was repeated before addition of HRP substrate solution (Pierce) for film exposure. Antibodies used to test for IRP1 expression that were not capable of recognizing the protein in HL60 lysates were from Sigma (Cat# HPA019371 & 536–550), Santa Cruz Biotechnology (sc166022), and Abcam (ab54718). The first antibody recognized native IRP1 in Hela lysates but only unknown fragments in HL60 lysates. Antibodies for DNA repair were obtained from Novus Biologicals # NB100-308 (ATR) and # NB100-504 (APE1), and from Santa Cruz Biotech #sc-5282 (DNA-PK). Beta actin control antibodies were obtained from Thermo Scientific #MA1-91399.

## Supporting Information

Figure S1 **Multiple IRP1-targeted shRNA constructs were capable of reducing IRP1 transcript levels and reducing gamma ray-induced apoptosis.** Panel a: mRNA was isolated from stably-expressing shRNA constructs and assayed for IRP1 transcript by qPCR. Data are mean +/− SEM from two independent experiments. Panel b: different constructs were irradiated with indicated doses of gamma rays and assayed for apoptosis 48 h post-IR by annexin V and PI staining. Data are mean +/− SEM from two independent experiments. * = p<0.05 for the least significant cell line relative to SCR by two-way ANOVA with Bonferroni post-tests.
(TIFF)

Figure S2 **Time-dependence of IR-induced apoptosis in HL60 cells.** Cells were irradiated with indicated doses of gamma rays and assayed for apoptosis at indicated times post-IR by annexin V & PI staining. Data are mean +/− SEM from three independent experiments.
(TIFF)

Figure S3 **Representative γH2A.X foci images in irradiated SCR and IRP1- cells.** Cells were treated with indicated doses of gamma IR and fixed at indicated times before staining for phosphorylated S139 H2A.X and imaged by confocal microscopy.
(TIF)

Figure S4 **Baseline cell cycle analysis indicates slightly higher percentage of cells containing 4n–8n DNA content in IRP1- cells.** Baseline cell cycle distributions were analyzed by PI content on fixed cells and analyzed by flow cytometry. Live cells were gated and plotted and PI height vs width content to determine cells with 2n–8n DNA content for SCR (panel a) and IRP1- (panel b) cells. Percentages of cells with 2n–4n DNA content are indicated.
(TIF)

Figure S5 **Baseline DNA repair protein expression does not differ in IRP1- and SCR cells.** 25 µg of cell lysates were run on polyacrylamide SDS-PAGE gels, transferred to PVDF membranes, and probed for indicated proteins by western blot.
(TIF)

Figure S6 **Late G2 checkpoint accumulation and duration is similar in IRP1- and SCR cells.** Panel a: Cells were irradiated with indicated doses of gamma IR and collected at 24 h post-IR and assayed for the number of cells in mitosis. Panel b: Cells were irradiated 6 Gy gamma rays and assayed for mitotic index at indicated timepoints following IR. Panel c: The percent of binucleated cells were quantified in greater than 500 cells per sample 20 h following 0 or 4 Gy of gamma rays. Panel d: percent micronuclei were quantified in at least 50 binucleated cells. Data are mean +/− SEM from two independent experiments.
(TIFF)

## References

[1] Hentze MW, Muckenthaler MU, Andrews NC (2004) Balancing acts: molecular control of mammalian iron metabolism. Cell 117: 285–297. 15109490 10.1016/s0092-8674(04)00343-5

[2] Kruszewski M (2003) Labile iron pool: the main determinant of cellular response to oxidative stress. Mutat Res 531: 81–92. 14637247 10.1016/j.mrfmmm.2003.08.004

[3] Rouault TA (2006) The role of iron regulatory proteins in mammalian iron homeostasis and disease. Nat Chem Biol 2: 406–414. 16850017 10.1038/nchembio807

[4] Walden WE, Selezneva AI, Dupuy J, Volbeda A, Fontecilla-Camps JC, et al (2006) Structure of dual function iron regulatory protein 1 complexed with ferritin IRE-RNA. Science 314: 1903–1908. 17185597 10.1126/science.1133116

[5] Karin M, Mintz B (1981) Receptor-mediated endocytosis of transferrin in developmentally totipotent mouse teratocarcinoma stem cells. J Biol Chem 256: 3245–3252. 6259157

[6] Levi S, Luzzago A, Cesareni G, Cozzi A, Franceschinelli F, et al (1988) Mechanism of ferritin iron uptake: activity of the H-chain and deletion mapping of the ferro-oxidase site. A study of iron uptake and ferro-oxidase activity of human liver, recombinant H-chain ferritins, and of two H-chain deletion mutants. J Biol Chem 263: 18086–18092. 3192527

[7] Volz K (2008) The functional duality of iron regulatory protein 1. Curr Opin Struct Biol 18: 106–111. 18261896 10.1016/j.sbi.2007.12.010 PMC2374851

[8] Wang W, Di X, D'Agostino RB Jr, Torti SV, Torti FM (2007) Excess capacity of the iron regulatory protein system. J Biol Chem 282: 24650–24659. 17604281 10.1074/jbc.M703167200

[9] Daly MJ, Gaidamakova EK, Matrosova VY, Vasilenko A, Zhai M, et al (2007) Protein oxidation implicated as the primary determinant of bacterial radioresistance. PLoS Biol 5: e92. 17373858 10.1371/journal.pbio.0050092 PMC1828145

[10] Daly MJ, Gaidamakova EK, Matrosova VY, Kiang JG, Fukumoto R, et al (2010) Small-molecule antioxidant proteome-shields in Deinococcus radiodurans. PLoS One 5: e12570. 20838443 10.1371/journal.pone.0012570 PMC2933237

[11] Krisko A, Radman M (2010) Protein damage and death by radiation in Escherichia coli and Deinococcus radiodurans. Proc Natl Acad Sci U S A 107: 14373–14377. 20660760 10.1073/pnas.1009312107 PMC2922536

[12] Krisko A, Leroy M, Radman M, Meselson M (2012) Extreme anti-oxidant protection against ionizing radiation in bdelloid rotifers. Proc Natl Acad Sci U S A 10.1073/pnas.1119762109 PMC3289372 22308443

[13] Sobota JM, Imlay JA (2011) Iron enzyme ribulose-5-phosphate 3-epimerase in Escherichia coli is rapidly damaged by hydrogen peroxide but can be protected by manganese. Proc Natl Acad Sci U S A 108: 5402–5407. 21402925 10.1073/pnas.1100410108 PMC3069151

[14] Imlay JA (2008) Cellular defenses against superoxide and hydrogen peroxide. Annu Rev Biochem 77: 755–776. 18173371 10.1146/annurev.biochem.77.061606.161055 PMC3057177

[15] Keyer K, Imlay JA (1996) Superoxide accelerates DNA damage by elevating free-iron levels. Proc Natl Acad Sci U S A 93: 13635–13640. 8942986 10.1073/pnas.93.24.13635 PMC19375

[16] Orino K, Lehman L, Tsuji Y, Ayaki H, Torti SV, et al (2001) Ferritin and the response to oxidative stress. Biochem J 357: 241–247. 11415455 10.1042/0264-6021:3570241 PMC1221947

[17] Epsztejn S, Glickstein H, Picard V, Slotki IN, Breuer W, et al (1999) H-ferritin subunit overexpression in erythroid cells reduces the oxidative stress response and induces multidrug resistance properties. Blood 94: 3593–3603. 10552971

[18] Goralska M, Holley BL, McGahan MC (2001) Overexpression of H- and L-ferritin subunits in lens epithelial cells: Fe metabolism and cellular response to UVB irradiation. Invest Ophthalmol Vis Sci 42: 1721–1727. 11431434

[19] MacKenzie EL, Ray PD, Tsuji Y (2008) Role and regulation of ferritin H in rotenone-mediated mitochondrial oxidative stress. Free Radic Biol Med 44: 1762–1771. 18325346 10.1016/j.freeradbiomed.2008.01.031 PMC2682214

[20] Singh A, Bodas M, Wakabayashi N, Bunz F, Biswal S (2010) Gain of Nrf2 function in non-small-cell lung cancer cells confers radioresistance. Antioxid Redox Signal 13: 1627–1637. 20446773 10.1089/ars.2010.3219 PMC3541552

[21] Franken NA, ten Cate R, Krawczyk PM, Stap J, Haveman J, et al (2011) Comparison of RBE values of high-LET alpha-particles for the induction of DNA-DSBs, chromosome aberrations and cell reproductive death. Radiat Oncol 6: 64. 21651780 10.1186/1748-717X-6-64 PMC3127784

[22] Barendsen GW (1994) The relationships between RBE and LET for different types of lethal damage in mammalian cells: biophysical and molecular mechanisms. Radiat Res 139: 257–270. 8073108

[23] Jakubowski W, Bartosz G (2000) 2,7-dichlorofluorescin oxidation and reactive oxygen species: what does it measure? Cell Biol Int 24: 757–760. 11023655 10.1006/cbir.2000.0556

[24] Blocher D (1988) DNA double-strand break repair determines the RBE of alpha-particles. Int J Radiat Biol 54: 761–771. 2902170 10.1080/09553008814552201

[25] Rogakou EP, Boon C, Redon C, Bonner WM (1999) Megabase chromatin domains involved in DNA double-strand breaks in vivo. J Cell Biol 146: 905–916. 10477747 10.1083/jcb.146.5.905 PMC2169482

[26] Galy B, Ferring-Appel D, Sauer SW, Kaden S, Lyoumi S, et al (2010) Iron regulatory proteins secure mitochondrial iron sufficiency and function. Cell Metab 12: 194–201. 20674864 10.1016/j.cmet.2010.06.007

[27] Belmokhtar CA, Hillion J, Segal-Bendirdjian E (2001) Staurosporine induces apoptosis through both caspase-dependent and caspase-independent mechanisms. Oncogene 20: 3354–3362. 11423986 10.1038/sj.onc.1204436

[28] Hahn SM, Tochner Z, Krishna CM, Glass J, Wilson L, et al (1992) Tempol, a stable free radical, is a novel murine radiation protector. Cancer Res 52: 1750–1753. 1551104

[29] Mitchell JB, DeGraff W, Kaufman D, Krishna MC, Samuni A, et al (1991) Inhibition of oxygen-dependent radiation-induced damage by the nitroxide superoxide dismutase mimic, tempol. Arch Biochem Biophys 289: 62–70. 1654848 10.1016/0003-9861(91)90442-l

[30] Campbell JL, Modrich P (2006) DNA repair. Amsterdam ; Boston: Elsevier/Academic Press.

[31] Hall EJ, Giaccia AJ (2012) Radiobiology for the radiologist. Philadelphia: Wolters Kluwer Health/Lippincott Williams & Wilkins. p. p.

[32] Daly MJ, Gaidamakova EK, Matrosova VY, Vasilenko A, Zhai M, et al (2004) Accumulation of Mn(II) in Deinococcus radiodurans facilitates gamma-radiation resistance. Science 306: 1025–1028. 15459345 10.1126/science.1103185

[33] Seideman JH, Stancevic B, Rotolo JA, McDevitt MR, Howell RW, et al (2011) Alpha Particles Induce Apoptosis through the Sphingomyelin Pathway. Radiat Res 10.1667/rr2472.1 PMC3185310 21631289

[34] Neti PV, de Toledo SM, Perumal V, Azzam EI, Howell RW (2004) A multi-port low-fluence alpha-particle irradiator: fabrication, testing and benchmark radiobiological studies. Radiat Res 161: 732–738. 15161346 10.1667/rr3181 PMC3040107

[35] Charlton DE, Sephton R (1991) A relationship between microdosimetric spectra and cell survival for high-LET irradiation. Int J Radiat Biol 59: 447–457. 1671694 10.1080/09553009114550401

[36] Theunissen J-WF, Petrini JHJ (2006) DNA Repair: Methods for Studying the Cellular Response to DNA Damage. Amsterdam; Boston: Elsevier/Academic Press. 266–270 p.

[37] Fenech M, Morley AA (1985) Measurement of micronuclei in lymphocytes. Mutat Res 147: 29–36. 3974610 10.1016/0165-1161(85)90015-9

[38] Du J, Gebicki JM (2004) Proteins are major initial cell targets of hydroxyl free radicals. Int J Biochem Cell Biol 36: 2334–2343. 15313477 10.1016/j.biocel.2004.05.012

