# Supplementary figures and images for: Dysregulation of IRP1-Mediated Iron Metabolism Causes Gamma Ray-specific Radioresistance in Leukemia Cells
Source: PLoS One. 2012 Nov 14;7(11):e48841. doi: 10.1371/journal.pone.0048841 (PMC3498264; doi:10.1371/journal.pone.0048841)

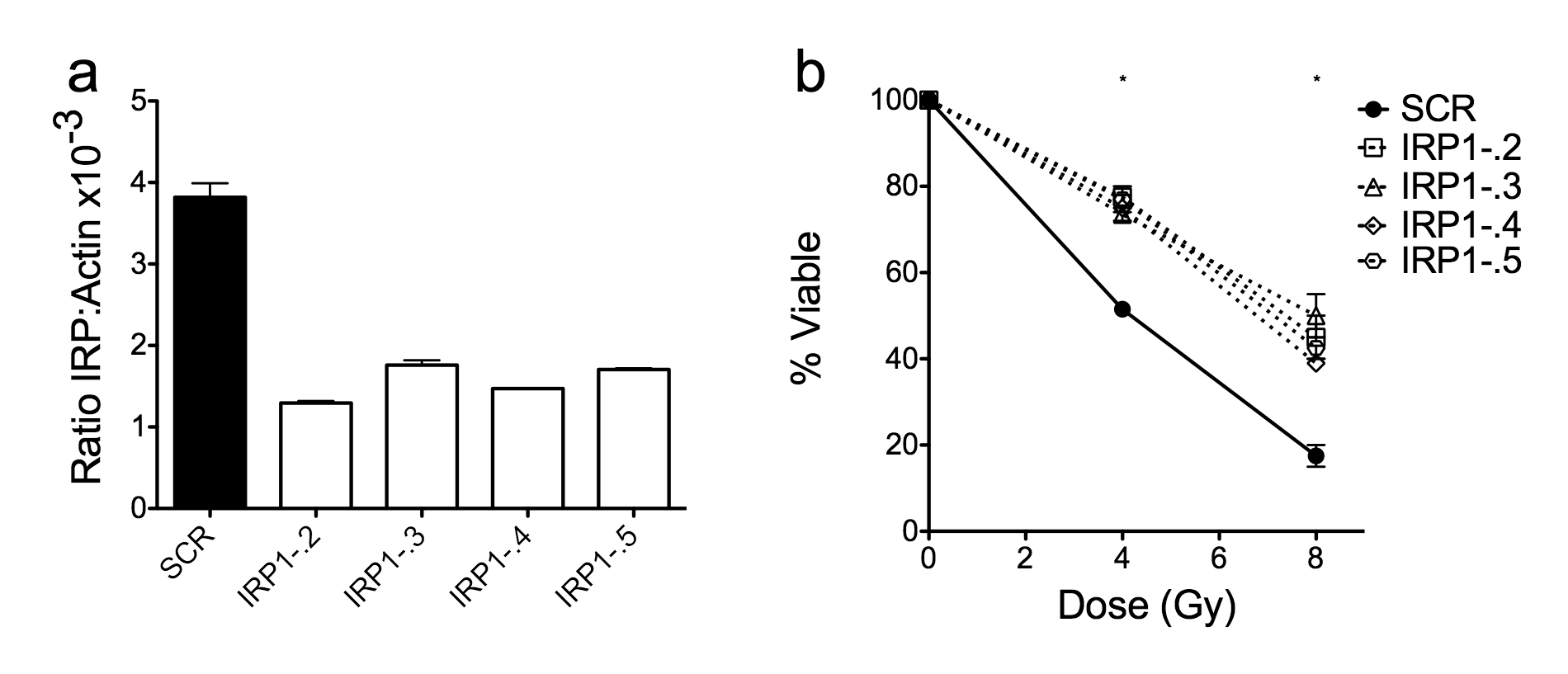

Supplement: Figure S1 — Multiple IRP1-targeted shRNA constructs were capable of reducing IRP1 transcript levels and reducing gamma ray-induced apoptosis. Panel a: mRNA was isolated from stably-expressing shRNA constructs and assayed for IRP1 transcript by qPCR. Data are mean +/− SEM from two independent experiments. Panel b: different constructs were irradiated with indicated doses of gamma rays and assayed for apoptosis 48 h post-IR by annexin V and PI staining. Data are mean +/− SEM from two independent experiments. * = p<0.05 for the least significant cell line relative to SCR by two-way ANOVA with Bonferroni post-tests. (TIFF) [file pone.0048841.s001.tiff]

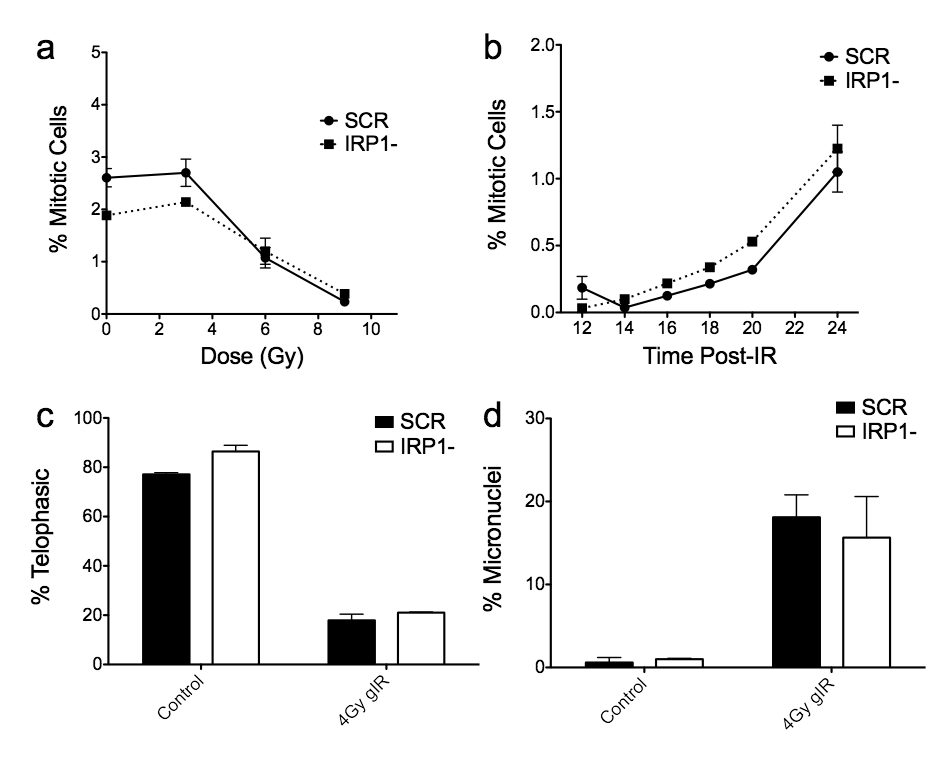

Supplement: Figure S2 — Time-dependence of IR-induced apoptosis in HL60 cells. Cells were irradiated with indicated doses of gamma rays and assayed for apoptosis at indicated times post-IR by annexin V & PI staining. Data are mean +/− SEM from three independent experiments. (TIFF) [file pone.0048841.s002.tiff]

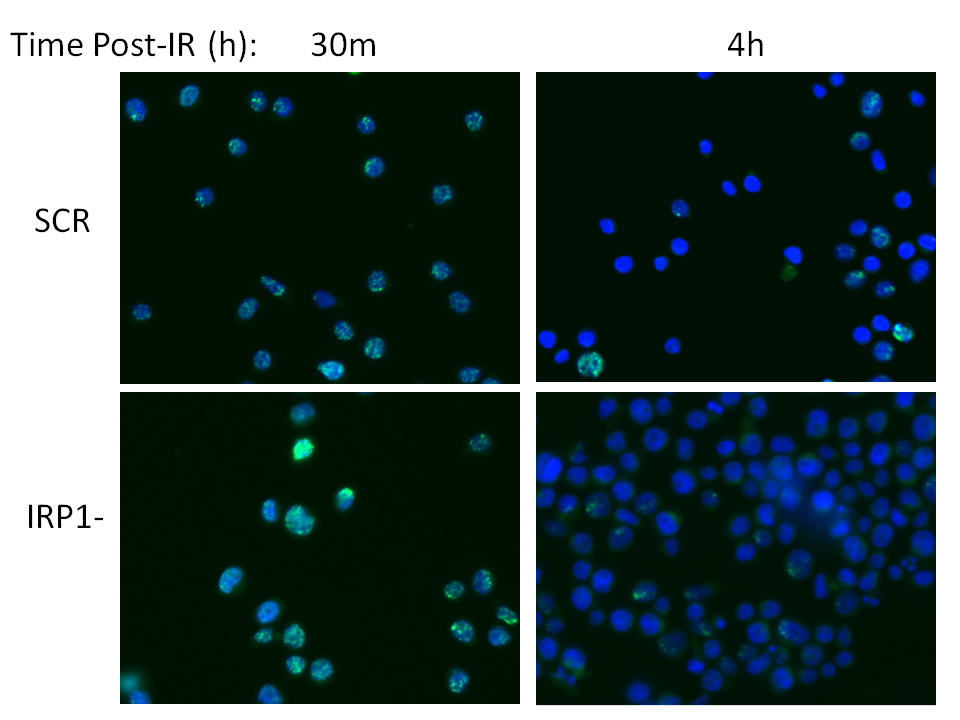

Supplement: Figure S3 — Representative γH2A.X foci images in irradiated SCR and IRP1- cells. Cells were treated with indicated doses of gamma IR and fixed at indicated times before staining for phosphorylated S139 H2A.X and imaged by confocal microscopy. (TIF) [file pone.0048841.s003.tif]

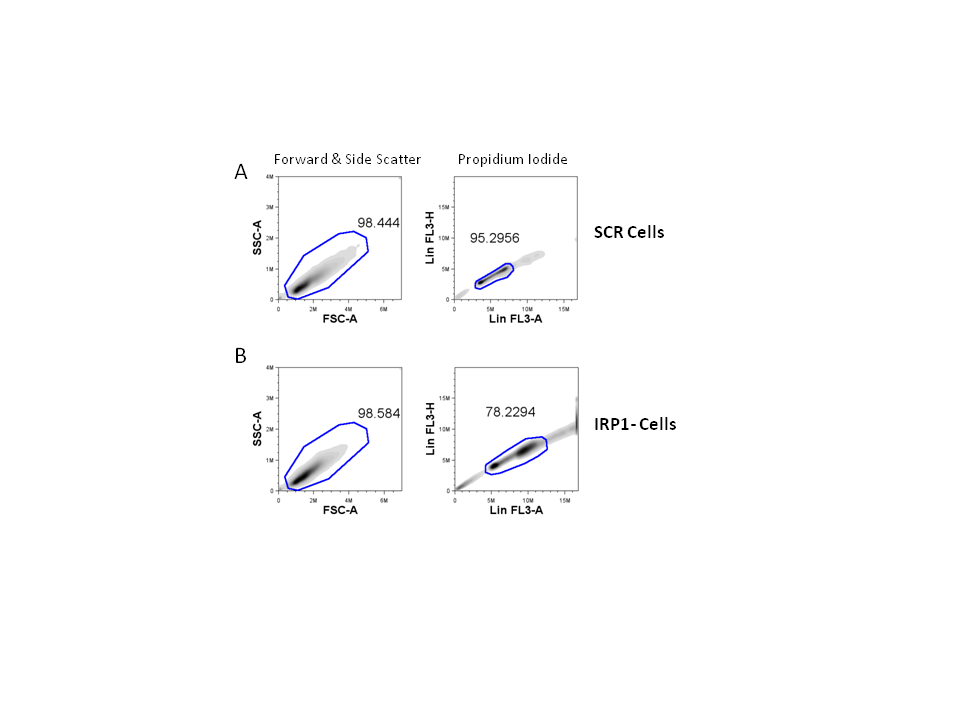

Supplement: Figure S4 — Baseline cell cycle analysis indicates slightly higher percentage of cells containing 4n–8n DNA content in IRP1- cells. Baseline cell cycle distributions were analyzed by PI content on fixed cells and analyzed by flow cytometry. Live cells were gated and plotted and PI height vs width content to determine cells with 2n–8n DNA content for SCR (panel a) and IRP1- (panel b) cells. Percentages of cells with 2n–4n DNA content are indicated. (TIF) [file pone.0048841.s004.tif]

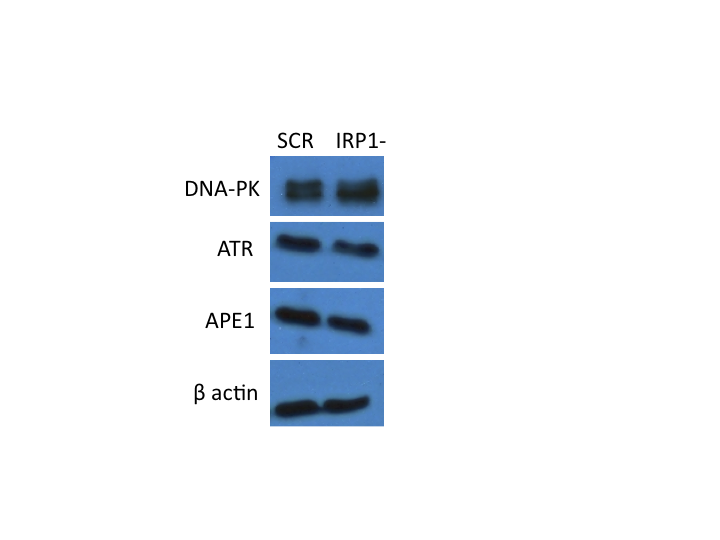

Supplement: Figure S5 — Baseline DNA repair protein expression does not differ in IRP1- and SCR cells. 25 µg of cell lysates were run on polyacrylamide SDS-PAGE gels, transferred to PVDF membranes, and probed for indicated proteins by western blot. (TIF) [file pone.0048841.s005.tif]

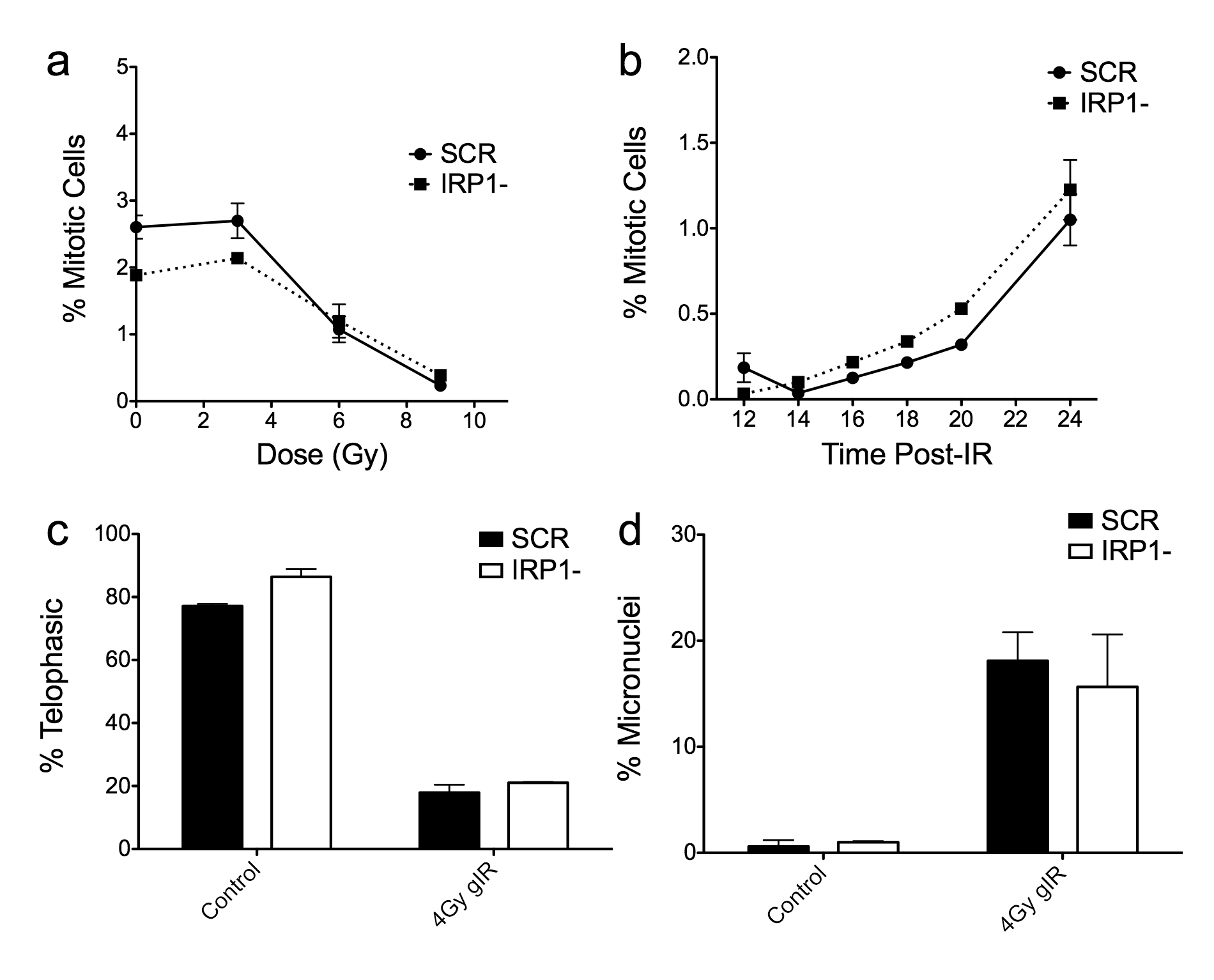

Supplement: Figure S6 — Late G2 checkpoint accumulation and duration is similar in IRP1- and SCR cells. Panel a: Cells were irradiated with indicated doses of gamma IR and collected at 24 h post-IR and assayed for the number of cells in mitosis. Panel b: Cells were irradiated 6 Gy gamma rays and assayed for mitotic index at indicated timepoints following IR. Panel c: The percent of binucleated cells were quantified in greater than 500 cells per sample 20 h following 0 or 4 Gy of gamma rays. Panel d: percent micronuclei were quantified in at least 50 binucleated cells. Data are mean +/− SEM from two independent experiments. (TIFF) [file pone.0048841.s006.tiff]
